# Combining Ability, Maternal Effects, and Heritability of Drought Tolerance, Yield and Yield Components in Sweetpotato

**DOI:** 10.3389/fpls.2016.01981

**Published:** 2017-01-10

**Authors:** Placide Rukundo, Hussein Shimelis, Mark Laing, Daphrose Gahakwa

**Affiliations:** ^1^African Centre for Crop Improvement, University of KwaZulu-NatalPietermaritzburg, South Africa; ^2^Rwanda Agriculture Board, HorticultureKigali, Rwanda

**Keywords:** canopy temperature, canopy wilting, clone, crosses, families, gene action

## Abstract

Knowledge on gene action and trait expression are important for effective breeding. The objective of this study was to determine the general combining ability (GCA), specific combining ability (SCA), maternal effects and heritability of drought tolerance, yield and yield components of candidate sweetpotato clones. Twelve genotypes selected for their high yield, dry matter content or drought tolerance were crossed using a full diallel mating design. Families were field evaluated at Masoro, Karama, and Rubona Research Stations of Rwanda Agriculture Board. Success rate of crosses varied from 1.8 to 62.5% with a mean of 18.8%. Family by site interaction had significant effect (*P* < 0.01) on storage root and vine yields, total biomass and dry matter content of storage roots. The family effects were significant (*P* < 0.01) for all parameters measured. Broad sense heritability estimates were 0.95, 0.84, 0.68, 0.47, 0.74, 0.75, 0.50, and 0.58 for canopy temperature (CT), canopy wilting (CW), root yield, skin color, flesh color, dry matter content, vine yield and total biomass, respectively. The GCA effects of parents and SCA effects of crosses were significant (*P* < 0.01) for CT, CW, storage root, vine and biomass yields, and dry matter content of storage root. The ratio of GCA/SCA effects for CT, CW, yield of storage roots and dry matter content of storage roots were higher than 50%, suggesting the preponderance of additive over non-additive gene action in the expression of these traits. Maternal effects were significant (*P* < 0.05) among families for CT, CW, flesh color and dry matter content, vine yield and total biomass. Across sites, the best five selected families with significant SCA effects for storage root yield were, Nsasagatebo × Otada 24, Otada 24 × Ukerewe, 4-160 × Nsasagatebo, K513261 × 2005-034 and Ukerewe × K513261 with 11.0, 9.7, 9.3, 9.2, 8.6 t/ha, respectively. The selected families are valuable genetic resources for sweetpotato breeding for drought tolerance, yield and yield components in Rwanda or similar environments.

## Introduction

Sweetpotato *(Ipomoea batatas* [L.] Lam; 2*n* = 6 × = 90) is an important root crop grown in more than 110 countries on an estimated area of 110 × 10^6^ ha with an annual production of 9000 metric tons (FAOSTAT, 2014). In most sub-Saharan Africa countries, it is widely grown in smallholder farmer systems across various agro-ecological zones, with excellent tolerance to various abiotic and biotic stresses. Sweetpotato has become the main staple food for many families in Uganda, Rwanda, and Burundi in Eastern Africa, where annual per capita consumption of fresh roots is above 80 kg (FAOSTAT, 2014). The storage roots of sweetpotato are rich in carbohydrates and its leaves are rich in proteins. Orange fleshed sweetpotato varieties are rich in β-carotene, a precursor of vitamin A, while purple fleshed sweetpotato varieties contain anthocyanin, which is a powerful anti-oxidant (Lebot, 2009). Sweetpotato flour can be used as a partial substitute of wheat flour in bakeries and pasta products, allowing for import substitute for wheat flour (Tan et al., 2007). However, yield and yield components, and quality traits of sweetpotato genotypes vary due to differences in genetic constitution, the environment and genotype-by-environment interactions.

An ideotype is determined by genetic components explained by gene action such as additive, dominance, epistatic or overdominance effects, and the environment in which it is grown (Fasoula and Fasoula, 2003). The magnitude and direction of genetic components are estimated through various parameters including combining ability, heritability and heterosis analyses. Knowledge of gene action and associated trait expression is important for effective breeding and selection (Grami et al., 1977; Ma-Teresa et al., 1994).

Combining ability analysis helps to identify superior parents to be used in breeding programs or to identify promising cross combinations for cultivar development (Acquaah, 2007). General combining ability (GCA) is directly related to the breeding value of a parent and is associated with additive genetic effects, while specific combining ability (SCA) is the relative performance of a cross that is associated with non-additive gene action, predominantly contributed by dominance, epistasis, or genotype × environment interaction effects (Rojas and Sprague, 1952; Falconer and Mackay, 1996). Therefore, both GCA and SCA effects are important in the selection or development of breeding populations (Viana and Matta, 2003).

The distribution of cytoplasmic genetic materials into gametes is unequal and unpredictable (Roach and Wulff, 1987; Acquaah, 2007). Grami and Stefansson (1977) reported that the maternal effects on protein and oil content in a summer rape seed crop observed during the F1 generation changed in the F2, due to inadequate distribution of cytoplasm genes during gamete formation. Hence, it is difficult to maintain the maternal effects in sexually reproducing crops. Maternal genetic effects can be maintained in vegetatively propagated crops such as sweetpotato, owing to the inherently identical propagation. Therefore, investigation and identification of maternal effects for desirable traits can be beneficial in breeding of sweetpotato, which may enhance responses to selection (Falconer and Mackay, 1996).

Heritability is categorized into broad sense heritability (H^2^) and narrow sense heritability (h^2^) and is a measure of the proportion of the genetic variance out of the total phenotypic variance present in a population. It shows the degree to which offspring can be expected to resemble their parents for a specific trait (Ma-Teresa et al., 1994; Sleper and Poehlman, 2006). When breeding clonally propagated species such as sweetpotato in which both additive and non-additive gene actions are fixed and transferred from parent to offspring, broad sense heritability is useful. However, in half sib families of sexually propagated crops, heritability in the narrow sense is important because alleles responsible for non-additive genetic variations are not fully recovered due to reshuffling of genes (Sleper and Poehlman, 2006). Selection of traits with low heritability could be enhanced through the use of controlled screening methods or controlled environments, molecular markers or selection based on breeding values (Gasura et al., 2010). Ma-Teresa et al. (1994) reported that the heritability of dry matter content in sweetpotato was 75–88% while Jones et al. (1986) and Lebot (2009) reported heritability levels for weight of storage roots of 61% for families and 59% for parentals (Jones et al., 1986; Lebot, 2009).

Genetic studies in sweetpotato are limited due to self- and cross-incompatibility, high level of polyploidy and limited flowering ability and seed setting (Lin et al., 2007). Knowledge of the genetics of sweetpotato traits is helpful for efficient selection and breeding. Development of sweetpotato varieties with complementary traits to satisfy the food demand and changing end-users' preferences is dependent on information of genetic attributes of parents and progenies. This, in turn, depends on the magnitude and direction of genetic effects on traits of economic interest. Therefore, the objective of this study was to determine general combining ability (GCA), specific combining ability (SCA), maternal effects and heritability of drought tolerance, yield and yield components of newly developed sweetpotato clones.

## Materials and methods

### Plant materials

Twelve selected sweetpotato genotypes described in Table 1 were included in the study to generate new genetic combinations. The parents were selected based on field, *in-vitro* and greenhouse evaluations aimed at flowering ability, yield potential, dry matter content of storage root or drought tolerance (Rukundo et al., 2015).

**Table 1:** **Description of sweetpotato parents used in crossing in the study**.

**No**	**Genotype name**	**Agronomic traits**	**Skin color**	**Flesh color**	**Origin**
1	2005-020	High yield	White	White	NARO
2	2005-034	High DMC	White	Orange	NARO
3	2005-110	High DMC	Yellow	Yellow	NARO
4	4-160	Drought tolerant	White	White	ISAR
5	8-1038	Drought tolerant	Red	White	ISAR
6	K513261	High yield	Red	White	IITA
7	Kwezikumwe	High yield	Yellow	Yellow	ISAR
8	Nsasagatebo	Drought tolerant	White	White	Landrace
9	Otada 24	High yield	Red	White	NARO
10	Purple 4419	Drought tolerant	Red	Orange	ISAR
11	SPK004	High DMC	Pink	Orange	KARI
12	Ukerewe	High DMC	Red	Orange	CIP

*DMC, Dry matter content; ISAR, Institut des Sciences Agronomiques du Rwanda; IITA, International Institute of Tropical Agriculture; KARI, Kenya Agriculture Research Institute; NARO, National Agricultural Research Organisation/Uganda; CIP, International Potato Center*.

### Crosses and mating design

**Figure 1:**
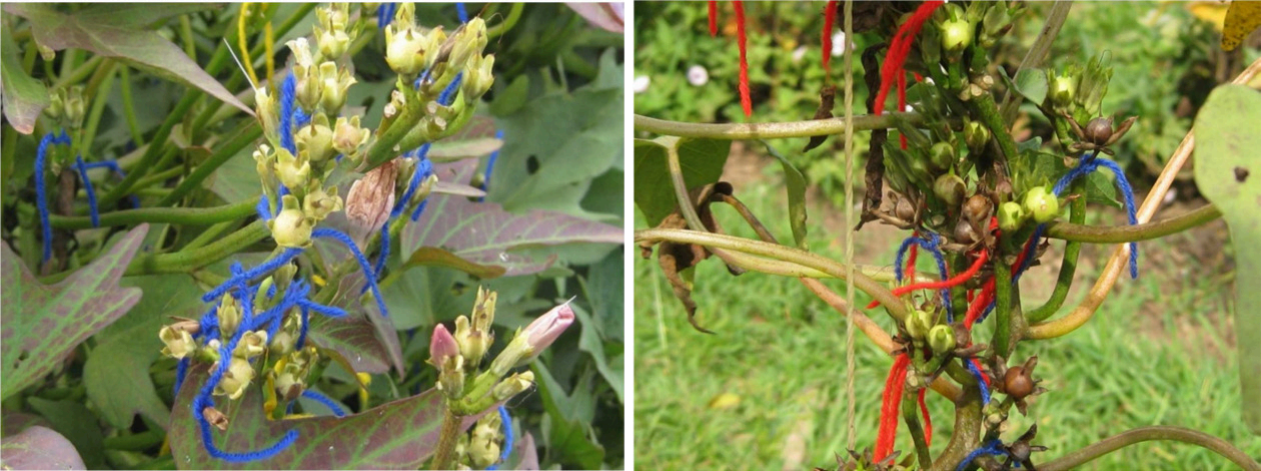
**Immature capsules (left) and mature capsules (right) of sweetpotato resulted from hand crosses**.

The 12 parents were crossed using a full diallel mating design. A crossing block was established between May 2013 and February 2014 at the Rubona Research Station of the Rwanda Agriculture Board (RAB). Plants were established in a well-prepared and mulched soil supplied with organic manure at planting. The crossing block was provided with supplemental irrigation twice a week. Vines were tended to grow on metallic trellises tied with plastic twine. Weeding and other agronomic practices were carried out when necessary. Flower buds that were near to open were closed with a piece of aluminum foil at about 4:00 pm. The next day each flower was hand pollinated between 6:00 and 9:00 a.m. Each pollinated flower was labeled and tagged (Figure 1). The dried capsules from successful crosses were regularly harvested, threshed and seed kept in a seed bag.

### Field establishment for evaluation of clonal families

**Figure 2:**
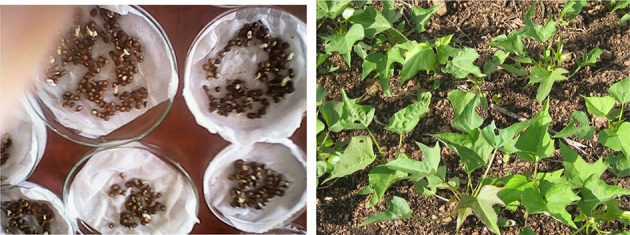
**Germination of sweetpotato seeds after scarification (left) and seedling plants (right)**.

Seeds harvested after successful crosses were scarified using the method described by Wilson et al. (1989) to induce germination. Briefly, seeds were soaked in concentrated sulphuric acid (98% H_2_SO_4_) using a vortex mixer for 40 min. The acid was discarded and seeds rinsed under running water for 10 min. Thereafter, seeds were placed in petri dishes lined with moistened filter paper and covered with cotton wool. The petri dishes were kept in the laboratory at ambient temperature. After 3 days, germinated seeds (Figure 2) were transplanted into a seedling nursery bed. Seedlings were used to provide vines for subsequent clonal evaluation trials.

Field trials were established in September 2014 at the Karama, Masoro and Rubona Research Stations of RAB. The climate and soil description of the sites are summarized in Table 2. Vine cuttings from 64 families (56 successful crosses and 8 parents) were planted in the field using an alpha lattice design with three replications. Cuttings with 4 to 5 nodes were planted with inter-row spacing of 80 cm and intra-row spacing of 50 cm. Experimental plots were bordered by growing two rows of a sweetpotato variety NASPOT 9 O. Weeding was carried out as required and no fertilizer and pesticide were applied. Harvesting was carried out 135 days after planting.

**Table 2:** **Geographic location, soil characteristics, temperature and rainfall of the Karama, Masoro and Rubona Research Station sites in Rwanda**.

**Parameters**	**Description**	**Karama**	**Masoro**	**Rubona**
Geographic coordinates	Latitude	S02°16′46.5″	S01°55′40.0″	S02°29′03.2″
	Longitude	E030°16′06.2″	E030°10′04.0″	E029°45′58.2″
	Altitude (m above sea level)	1330	1482	1673
Soil	Types	Sandy and clay soils	Clay and kaolin soils	Clay and kaolin soils
Temperature (°C)	Minimum	17.2	15.7	13.4
	Maximum	28.4	27.1	26.9
Rain fall (mm)	Sept 2014- Feb 2015	567.9	722.4	804.3

### Data collection

#### Success rate of crosses

The number of successful crosses carried out was recorded periodically and during harvesting. These data were used to determine success rate of crosses and compatibility between the selected parents. The mean number of viable seeds per capsule was recorded. The percentage of seed germination was determined after seed scarification before planting in soil.

#### Agronomic data

Drought tolerance among clonal families and parents were assessed using canopy temperature (CT) measured with an infrared thermometer (Major Tech, MT694) and canopy wilting (CW) data collected at the Karama site. CT and WT were recorded during sunny days between 12h00 and 15h00. CT was rated using a 1 to 5 scale where, score 1: no wilting, 2: few top leaves showed wilting, 3: half of the leaves showed wilting, 4: severe wilting, about 75% of the leaves showed wilting and 5: severely wilted and plant death (Blum, 2002). Fresh weight of storage root and vine yields, skin and flesh characteristics of storage root were recorded using the standard descriptors developed by CIP (Huamán, 1999). Furthermore, the dry matter content (DMC) was determined following the methods described by Carey and Reynoso (1996) and Tairo et al. (2008) with minor modifications. Briefly, two samples of 50 to 60 g were collected from the biggest, healthy storage roots of each clone and kept in a paper bag. These samples were dried in an oven at 70°C for 72 h. Dried samples were weighed with an analytical balance and the dry matter content was determined using the formula: Dry matter content (DM) % = [(Dry weight/Fresh weight) × 100].

### Data analysis

#### Success rate of crosses, number of seeds per capsule and germination

The success rate (%) of crosses was determined as a ratio of number of harvested seed capsules per total number of crosses carried out. The number of seeds per capsule was determined and averaged for each family. The germination rate was determined as the ratio of germinated seed to total number of seed scarified and planted for each family.

#### Analysis of variance

Plot yield data of storage root, vine and total biomass were converted to t ha^−1^. Data on yield, dry matter content of storage root and leaf temperatures were subjected to analysis of variance using the GLM procedure of the SAS 9.2 statistical program (SAS, 2004). When significant differences were detected, means were separated using the LSD test procedure at the 5% significance level (Cochran and Cox, 1992). The qualitative data of leaf wilting, skin color and fresh color were analyzed using the non-parametric Krusal-Wallis test procedure of the SPSS computer package (PASW statistics 18.0) (SPSS, 2006).

#### Estimation of general and specific combining ability effects and heritability

Analysis of variance was performed using the DIALLEL-SAS05 program (Zhang et al., 2005) to identify the significant level of general combining ability (GCA) of parents and specific combining ability (SCA) of crosses. The diallel analysis was performed using Griffing's (1956) Method 1 Model 2, with the genetic statistical model of:

(1) $$\begin{array}{r} {\text{Y}}_{\text{ij}}=\mu +{\text{g}}_{\text{i}}+{\text{g}}_{\text{j}}+{\text{s}}_{\text{ij}}+{\text{r}}_{\text{ij}}+{\text{b}}_{\text{k}}+{\text{e}}_{\text{ijkl}} \end{array}$$

Where: Y_ij_ = observed value of the cross between parent i and j; μ = overall mean; g_i_ = GCA effect of parent i; g_j_ = GCA effect of parent j; s_ij_ = SCA of the cross between parents i and j; r_ij_: reciprocal effect involving the reciprocal crosses between the i^th^ and j^th^ parents, b_k_ = effect of the k^th^ block; e_ijkl_ = experimental error associated with the ijkl^th^ individual observation, I, j = 1…, p: number of parents, k = 1…, a: number of blocks, l = 1…, c: number of replications.

The relative importance of GCA and SCA effects for each trait was determined following the general predicted ratio (GPR): GCA/SCA = 2 MSGCA/(2MSGCA + MSSCA) (Baker, 1978). The broad sense heritability (H^2^) of the above traits was determined using the following formula:

(2) $$\begin{array}{r} {\text{H}}^{2}=\text{V}{\text{g}}_{/}{\text{V}}_{\text{p}} \end{array}$$

Where H^2^: broad sense heritability, V_g_: genetic variance and V_p_: Phenotypic variance (Ma-Teresa et al., 1994; Acquaah, 2007).

## Results

### Compatibility among 12 selected sweetpotato genotypes

Initially 12 parents were selected and included for full diallel crosses. However, complete incompatibility of both direct and reciprocal crosses was observed among the following pairs: 2005-110 × 2005-034, 4-160 × 2005-020, 4-160 × 2005-034, Kwezikumwe × 2005-034, Kwezikumwe × 4-160, SPK004 × 2005-034, SPK004 × 4-160. The proportion of compatible crosses chosen among the 8 parents are summarized in Table 3. Partial incompatibility was observed in crosses involving Otada 24 × 2005-034 and Ukerewe × Nsasagatebo (Table 3). The success rate of crosses varied from 1.8% (8-1038 × Ukerewe) to 62.5% (Ukerewe × K513261) with a mean of 18.8% (Table 3). The best cross combinations with success rates of >45% were achieved in crosses between K513261 × 8-1038, 2005-034 × 8-1038 and 2005-034 × 8-1038. The number of seeds per capsule varied from about 1 (Otada 24 × 8-1038) to 3 (2005-034 × Ukerewe) with a mean of 1.6 (Table 3). About 3 seeds per capsule resulted from the following crosses: 2005-020 × Nsasagatebo, Ukerewe × 2005-020, 2005-034 × Ukerewe and Nsasagatebo × 8-1038. The germination rate varied from 0% (Ukerewe × 4-160, Nsasagatebo × 8-1038) to 85.1% (2005-020 × 2005-034) with a mean of 41.0% (Table 3).

**Table 3:** **Compatibility and success rate of crosses with corresponding number of seeds per capsule, and germination rate of scarified seeds**.

**No**	**Direct crosses**	**Compatibility**	**Success rate (%)**	**Seed/capsule**	**Germination (%)**
1	2005-034 × 2005-020	Compatible	16.8	1.9	37.5
2	4-160 × 2005-020	Compatible	15.4	1.5	38.5
3	4-160 × 2005-034	Incompatible	0.0	0.0	0.0
4	8-1038 × 2005-020	Compatible	3.7	1.8	53.8
5	8-1038 × 2005-034	Compatible	6.9	2.5	37.5
6	8-1038 × 4-160	Compatible	27.0	1.8	30.0
7	K513261 × 2005-020	Compatible	41.2	0.9	53.1
8	K513261 × 2005-034	Incompatible	0.0	0.0	0.0
9	K513261 × 4-160	Incompatible	0.0	0.0	0.0
10	K513261 × 8-1038	Compatible	47.2	1.2	67.5
11	Nsasagatebo × 2005-020	Compatible	15.2	1.1	20.8
12	Nsasagatebo × 2005-034	Compatible	10.5	1.7	10.0
13	Nsasagatebo × 4-160	Compatible	40.5	1.2	16.2
14	Nsasagatebo × 8-1038	Compatible	8.3	3.0	0.0
15	Nsasagatebo × K513261	Compatible	19.0	1.7	72.4
16	Otada 24 × 2005-020	Compatible	6.3	1.2	64.2
17	Otada 24 × 2005-034	Incompatible	0.0	0.0	0.0
18	Otada 24 × 4-160	Compatible	10.3	2.7	47.4
19	Otada 24 × 8-1038	Compatible	11.4	0.9	59.5
20	Otada 24 × K513261	Compatible	15.2	2.6	46.7
21	Otada 24 × Nsasagatebo	Compatible	38.6	1.7	67.6
22	Ukerewe × 2005-020	Compatible	30.2	2.8	66.7
23	Ukerewe × 2005-034	Compatible	18.6	1.5	50.0
24	Ukerewe × 4-160	Compatible	14.3	1.5	0.0
25	Ukerewe × 8-1038	Compatible	27.5	2.5	82.9
26	Ukerewe × K513261	Compatible	62.5	2.5	48.4
27	Ukerewe × Nsasagatebo	Incompatible	0.0	0.0	0.0
28	Ukerewe × Otada 24	Compatible	38.2	1.5	30.0
**No**	**Reciprocal crosses**	**Compatibility**	**Success rate (%)**	**Seed/capsule**	**Germination (%)**
29	2005-020 × 2005-034	Compatible	13.7	1.0	85.1
30	2005-020 × 4-160	Compatible	12.8	2.0	49.4
31	2005-034 × 4-160	Incompatible	0.0	0.0	0.0
32	2005-020 × 8-1038	Compatible	28.6	2.3	40.0
33	2005-034 × 8-1038	Compatible	47.8	1.5	72.0
34	4-160 × 8-1038	Compatible	11.4	1.6	45.7
35	2005-020 × K513261	Compatible	28.5	1.0	25.7
36	2005-034 × K513261	Incompatible	0.0	0.0	0.0
37	4-160 × K513261	Incompatible	0.0	0.0	0.0
38	8-1038 × K513261	Compatible	29.8	1.8	47.7
39	2005-020 × Nsasagatebo	Compatible	18.3	2.7	53.3
40	2005-034 × Nsasagatebo	Compatible	18.5	1.7	48.8
41	4-160 × Nsasagatebo	Compatible	37.2	1.4	52.2
42	8-1038 × Nsasagatebo	Compatible	29.3	1.5	30.6
43	K513261 × Nsasagatebo	Compatible	44.6	1.5	14.6
44	2005-020 × Otada 24	Compatible	14.1	1.3	68.8
45	2005-034 × Otada 24	Compatible	20.9	1.5	67.7
46	4-160 × Otada 24	Compatible	36.0	1.3	47.0
47	8-1038 × Otada 24	Compatible	13.2	1.8	14.3
48	K513261 × Otada 24	Compatible	11.8	1.5	20.0
49	Nsasagatebo × Otada 24	Compatible	7.8	1.4	50.0
50	2005-020 × Ukerewe	Compatible	43.5	1.8	51.4
51	2005-034 × Ukerewe	Compatible	11.9	2.9	72.7
52	4-160 × Ukerewe	Compatible	10.6	1.5	78.4
53	8-1038 × Ukerewe	Compatible	1.8	1.9	73.2
54	K513261 × Ukerewe	Compatible	13.4	1.4	47.1
55	Nsasagatebo × Ukerewe	Compatible	4.8	1.0	66.7
56	Otada 24 × Ukerewe	Compatible	15.4	1.5	70.0
	Mean		18.8	1.6	41.0

### Analysis of variance

A separate analysis of variance of each site showed significant differences among tested families for all parameters measured (data not presented). The combined analysis of variance revealed significant interactions (*P* ≤ 0.01) of family by site effect for storage root and vine yields, total biomass, and dry matter content of storage roots. A non-significant family by site interaction effect was detected for skin and flesh color of storage roots (Table 4). The family effects were significant (*P* ≤ 0.01) for all parameters evaluated (Table 4). The effects of sites were significantly different at *P* ≤ 0.05 for storage root and vine yields, total biomass, and dry matter content (Table 4). Overall, the family effects made a more significant contribution to the total variability than sites, and family by sites effects as shown by having the highest sum of squares for all evaluated traits (Table 4). Broad sense heritability (H^2^) values of 0.95, 0.84 0.68, 0.47, 0.74, 0.50, 0.58, and 0.75 were estimated for canopy temperature, canopy wilting, storage root yield, skin color, flesh color, yield of vine, total biomass and dry matter content, respectively (Table 4).

**Table 4:** **Mean squares and significant tests summarized from a combined analysis of variance of canopy temperature, canopy wilting, yield of storage roots, skin and flesh color, yield of vines, total biomass and dry matter content of storage roots of 64 sweetpotato families evaluated at three sites in Rwanda**.

**Source of variation**	**DF**	**Traits and mean squares**							
		**CT**	**CW**	**Storage root**	**Skin color**	**Flesh color**	**Vine yield**	**Biomass**	**DMC**
Site	2	64.68^**^	51.82^**^	606.98^*^	186.1 ns	2.08 ns	59597.94^**^	65617.55^**^	3214.97^**^
Rep (Site)	6	62.28ns	4.47 ns	577.78 ns	83.57 ns	1.01 ns	3160.25 ns	5428.14	545.08^**^
Family	63	31658.56^**^	982.45^**^	20534.25^**^	1456.59^**^	231.31^**^	174857.82^**^	286096.53^**^	76232.05^**^
Site × Family	126	1428.54 ns	129.61 ns	8274.97^**^	1267.96 ns	21.98 ns	114054.01^**^	134981.53^**^	22069.69^**^
H2		0.95	0.84	0.68	0.47	0.74	0.50	0.58	0.75

DF, degree of freedom; CT, canopy temperature; CW, canopy wilting; H^2^, broad sense heritability; DMC, Dry matter content;

** *Significant at p < 0.01*;

* *Significant at p < 0.05*;

*^ns^not significant)*.

### Field performance of families and parents

#### Canopy temperature and wilting

Experimental clones that showed the lowest CT (<20°C) were 4-160 × Ukerewe, 4-160 × Nsasagatebo, 8-1038 × 4-160, 4-160 × 8-1038, 8-1038 × 2005-020 and Nsasagatebo × Ukerewe. These clones had CT values of 18.9, 19.2, 19.3, 19.4, 19.7, and 19.8°C, respectively (Table 5). The parental genotypes Nsasagatebo, 4-160 and 8-1038 selected for their known drought tolerance, ranked among the best 10 with the lowest CT values measured at 17.8, 18.2, and 18.4°C, in that order (Table 5). The lowest CW values of 0.9, 1.1, 1.2, 1.3, 1.4, and 1.50 were observed in the following crosses: 4-160 × Nsasagatebo, 4-160 × Ukerewe, Nsasagatebo × 2005-020, Otada 24 × Nsasagatebo, 4-160 × K513261 and K513261 × 4-160, respectively (Table 5). Among the parents, Nsasagatebo showed the lowest CW (1.1), followed by 4-160 (1.5) and 8-1038 (1.6). High levels of CW of 4.0 to 4.3 were found in the families Otada 24 × 2005-020, 2005-034 × Nsasagatebo, Nsasagatebo × 2005-034 and 2005-020 × K513261 (Table 5).

**Table 5:** **Mean canopy temperature (CT), canopy wilting (CW), yields of storage roots and dry matter content (DMC) of storage roots of families and parents of sweetpotato clones evaluated across three sites in Rwanda**.

**Clones**	**CT (°C)**	**CW**	**Storage root yield (t ha–1)**			**Mean (t/ha)**	**DMC (%)**			**Mean (%)**
	**Karama**		**Karama**	**Masoro**	**Rubona**		**Karama**	**Masoro**	**Rubona**	
**DIRECT CROSSES**										
2005-020 × 2005-034	20.0	3.3	0.8	0.6	6.1	2.5	26.3	31.9	31.8	26.7
2005-020 × 4-160	21.0	3.8	1.7	2.8	3.4	2.6	32.8	35.2	33.6	33.9
2005-020 × 8-1038	20.7	3.4	0.0	0.9	1.5	1.2	NA	32.8	34.2	33.5
2005-020 × K513261	22.0	4.3	4.0	5.5	4.4	4.6	28.6	36.0	31.7	32.1
2005-020 × Nsasagatebo	23.0	4.1	0.0	0.0	0.0	0.0	NA	NA	NA	NA
2005-020 × Otada 24	20.8	3.5	1.6	1.4	8.1	3.7	28.7	34.7	33.4	29.0
2005-020 × Ukerewe	21.5	3.9	4.9	7.2	13.1	8.4	32.3	36.4	33.2	34.0
2005-034 × 4-160	0.0	0.0	0.0	0.0	0.0	0.0	NA	NA	NA	NA
2005-034 × 8-1038	21.4	3.2	6.3	3.9	6.3	5.5	28.6	29.9	32.4	30.3
2005-034 × K513261	21.8	2.8	6.4	5.0	8.4	6.6	32.0	35.2	34.8	34.0
2005-034 × Nsasagatebo	23.1	4.1	0.4	3.5	5.3	3.0	26.2	NA	32.2	29.2
2005-034 × Otada 24	0.0	0.0	0.0	1.9	2.3	2.1	0.0	39.8	28.0	33.0
2005-034 × Ukerewe	21.4	3.4	0.0	0.0	2.1	2.1	NA	NA	35.2	35.2
4-160 × 8-1038	19.3	3.7	6.4	3.1	6.7	5.4	29.8	33.7	35.1	32.9
4-160 × K513261	22.8	1.4	6.9	4.4	10.8	7.4	26.4	35.3	33.8	31.8
4-160 × Nsasagatebo	19.2	0.9	9.8	0.9	17.3	9.3	34.5	37.8	32.9	35.1
4-160 × Otada 24	21.7	3.1	2.1	0.0	1.9	2.0	27.2	0.0	33.7	30.0
4-160 × Ukerewe	18.9	1.1	1.1	4.9	3.2	3.0	29.1	35.3	24.2	26.2
8-1038 × K513261	23.1	2.8	0.0	13.3	6.3	6.5	NA	40.6	36.8	37.5
8-1038 × Nsasagatebo	20.8	3.8	0.0	0.0	2.8	2.8	NA	NA	33.8	33.8
8-1038 × Otada 24	23.6	3.0	0.0	0.0	0.0	0.0	NA	NA	NA	NA
8-1038 × Ukerewe	20.1	2.8	9.9	2.6	8.3	6.9	29.9	37.2	26.7	31.3
K513261 × Nsasagatebo	21.8	3.6	16.8	2.8	4.2	7.9	24.5	33.8	37.4	27.4
K513261 × Otada 24	21.2	2.8	4.8	8.4	9.9	7.7	28.4	32.1	33.7	31.4
K513261 × Ukerewe	20.8	2.7	0.0	3.5	12.3	5.3	NA	26.1	34.3	30.2
Nsasagatebo × Otada 24	20.0	3.8	18.9	9.8	1.4	11.0	26.5	34.6	32.4	27.8
Nsasagatebo × Ukerewe	19.8	3.8	0.0	0.0	5.6	5.6	NA	NA	35.3	35.3
Otada 24 × Ukerewe	21.8	3.8	6.4	5.1	17.6	9.7	28.7	36.5	32.0	32.4
**RECIPROCAL CROSSES**										
2005-034 × 2005-020	21.0	2.3	5.0	7.2	6.9	6.4	29.2	32.2	30.7	30.7
4-160 × 2005-020	20.9	2.6	6.0	6.3	8.2	6.8	27.0	29.3	31.3	29.2
4-160 × 2005-034	0.0	0.0	0.0	0.0	0.0	0.0	NA	NA	NA	NA
8-1038 × 2005-020	19.7	3.1	2.9	2.4	3.6	3.0	22.6	29.0	28.2	26.6
8-1038 × 2005-034	22.1	3.4	8.9	2.6	12.4	7.9	30.0	35.0	33.8	32.9
8-1038 × 4-160	19.4	1.7	7.9	5.1	5.4	6.1	32.9	35.5	32.6	33.6
K513261 × 2005-020	22.0	2.8	5.0	0.7	4.2	1.6	23.7	34.2	30.2	29.4
K513261 × 2005-034	22.4	3.2	11.9	2.0	13.6	9.2	33.8	36.4	31.4	33.9
K513261 × 4-160	21.7	1.5	6.0	0.8	4.5	3.8	27.6	33.3	33.6	31.5
K513261 × 8-1038	20.3	2.2	0.0	0.0	0.0	0.0	NA	NA	NA	NA
Nsasagatebo × 2005-020	21.5	1.2	3.0	4.0	3.2	3.4	23.9	33.6	31.7	29.7
Nsasagatebo × 2005-034	22.2	4.2	5.1	4.2	1.8	2.0	28.2	31.2	35.5	25.9
Nsasagatebo × 4-160	20.7	3.1	0.0	0.0	0.0	0.0	NA	NA	NA	NA
Nsasagatebo × 8-1038	21.2	3.3	0.0	0.0	16.8	5.6	NA	NA	37.2	37.2
Nsasagatebo × K513261	21.5	3.4	0.0	2.8	4.3	3.5	NA	35.7	33.9	34.5
Otada 24 × 2005-020	22.8	4.0	1.6	3.4	6.0	3.7	29.9	31.1	31.8	30.9
Otada 24 × 2005-034	23.0	3.1	0.0	0.0	0.0	0.0	NA	NA	NA	NA
Otada 24 × 4-160	0.0	0.0	10.4	3.4	8.8	7.5	34.4	31.5	31.2	35.7
Otada 24 × 8-1038	20.8	2.2	0.7	7.1	5.7	4.5	29.1	37.0	35.0	33.7
Otada 24 × K513261	20.8	1.9	7.1	6.8	7.4	7.1	28.1	31.9	34.5	31.5
Otada 24 × Nsasagatebo	20.0	1.3	0.0	0.0	2.8	2.8	NA	NA	34.0	34.0
Ukerewe × 2005-020	21.4	3.3	2.1	1.8	6.5	3.5	28.7	37.7	32.4	32.9
Ukerewe × 2005-034	22.0	3.1	4.6	5.6	7.0	5.7	35.8	38.9	33.8	31.5
Ukerewe × 4-160	21.9	3.5	0.0	0.0	0.0	0.0	NA	NA	NA	NA
Ukerewe × 8-1038	20.1	3.7	9.9	4.9	10.4	8.4	31.2	37.0	32.3	33.5
Ukerewe × K513261	22.2	3.2	5.2	10.4	10.3	8.6	26.3	36.0	32.9	31.8
Ukerewe × Nsasagatebo	21.9	2.8	0.0	0.0	0.0	0.0	NA	NA	NA	NA
Ukerewe × Otada 24	22.3	3.2	5.6	7.9	6.5	6.7	25.9	38.4	33.9	29.4
**PARENTS**										
2005-020	22.9	3.3	16.8	4.2	2.1	7.7	31.0	28.2	30.5	29.9
2005-034	20.3	4.0	18.9	9.8	9.8	12.8	32.8	40.5	34.3	35.8
4-160	18.2	1.5	19.6	12.6	9.1	13.8	36.0	42.5	32.7	37.1
8-1038	18.4	1.6	29.4	18.2	22.4	23.3	29.2	29.8	31.7	30.2
K513261	22.1	3.8	17.5	14.0	15.4	15.6	33.1	37.1	31.9	34.0
Nsasagatebo	17.9	1.1	8.4	4.2	14.7	9.1	35.0	39.5	33.0	35.8
Otada 24	22.2	3.6	14.7	12.6	11.9	13.1	35.1	40.1	30.3	35.1
Ukerewe	21.1	2.5	46.2	30.8	29.4	21.5	36.3	40.3	30.4	35.7
Average	17.0	2.7	6.5	4.5	6.9	5.7	22.4	26.0	28.1	25.5
LSD (5%)	4.1	1.4	19.7	5.5	10.5	13.2	14.6	2.2	3.1	8.7
CV (%)	1.3	2.2	11.0	6.2	28.8	16.0	8.4	0.1	3.1	3.3

*CT, canopy temperature; CW, canopy wilting; DMC, dry matter content of storage roots; NA, Not applicable*.

#### Storage root yields

The highest overall mean storage root yield of 6.9 t ha^−1^ was observed at Rubona site followed by the Karama and Masoro sites with 6.5 and 4.5 t ha^−1^, respectively (Table 5). At the Karama site, the five families showing the highest yields of storage roots were Nsasagatebo × Otada 24 (18.9 t ha^−1^), K513261 × Nsasagatebo (16.8 t ha^−1^), K513261 × 2005-034 (11.9 t ha^−1^), Otada 24 × 4-160 (10.4 t ha^−1^) and Ukerewe × 8-1038 (9.9 t ha^−1^). The best yielding families at Masoro were 8-1038 × K513261 (13.3 t ha^−1^), Ukerewe × K513261 (10.4 t ha^−1^), Nsasagatebo × Otada 24 (9.8 t ha^−1^), K513261 × Otada 24 (8.4 t ha^−1^), and Ukerewe × Otada 24 (7.9 t ha^−1^) (Table 5). At the Rubona site, the best five families for storage root yield were Otada 24 × Ukerewe (17.6 t ha^−1^), 4-160 × Nsasagatebo (17.3 t ha^−1^), Nsasagatebo × 8-1038 (16.8 t ha^−1^), K513261 × 2005-034 (13.6 t ha^−1^) and 2005-020 × Ukerewe (13.5 t ha^−1^) (Table 5). Across the three sites, the best five families for storage root yield were Nsasagatebo × Otada 24, Otada 24 × Ukerewe, 4-160 × Nsasagatebo, K513261 × 2005-034 and Ukerewe × K513261 with yields of 11.0, 9.7, 9.3, 9.2, and 8.6 t ha^−1^, respectively (Table 5). The parental genotypes used in these crosses exhibited mean storage root yields of 7.7 to 23.3 t ha^−1^. The parents, 8-1038 and Ukerewe were the best yielders producing 23.3 and 21.5 t ha^−1^, respectively (Table 5).

#### Dry matter content of storage roots

The highest mean dry matter content of storage roots (28.1%) was recorded at the Rubona site. At Masoro, the mean DMC was 26.0% while Karama had 22.4% (Table 5). At the Karama site, families such as Ukerewe × 2005-034, 4-160 × Nsasagatebo, Otada 24 × 4-160, K513261 × 2005-034, 8-1038 × 4-160 generated the highest DMC values of 35.8, 34.5, 34.4, 33.8 and 32.9 %, respectively (Table 5). The best DMC of 40.6, 39.8, 38.9, 38.4, 37.8% were observed at the Masoro site in the families of 8-1038 × K513261, 2005-034 × Otada 24, Ukerewe × 2005-034, Ukerewe × Otada 24 and 4-160 × Nsasagatebo, respectively (Table 5). At Rubona site, the best five families were K513261 × Nsasagatebo (37.4%), Nsasagatebo × 8-1038 (37.2%), 8-1038 × K513261 (36.7%), Nsasagatebo × 2005-034 (35.5%) and Nsasagatebo × Ukerewe (35.3%) (Table 5). Across all the study sites, the five best performing families were 8-1038 × K513261, Nsasagatebo × 8-1038, Otada 24 × 4-160, Nsasagatebo × Ukerewe and 4-160 × Nsasagatebo with DMC values of 37.5, 37.2, 35.7, 35.3 and 35.1%, respectively (Table 5). Overall, the parental clones, Ukerewe, 2005-034, Nsasagatebo, Otada 24, 4-160, K513261, 2005-020 and 8-1038 displayed high DMC values of 37.3, 35.8, 35.8, 35.1, 34.4, 34.0, 31.9, and 30.2%, respectively (Table 5).

#### Combining ability and maternal effects

Parentals and families had highly significant (*P* ≤ 0.01) general combining ability (GCA) and specific combining ability (SCA) effects, respectively, for CT, CW, yields of storage roots and vines, total biomass and dry matter content (DMC) of storage roots (Table 6). The ratios of GCA/SCA effect were >0.5 for CT, CW, yield of storage roots and DMC of storage roots, suggesting the predominance of additive over non-additive genetic effects. This ratio was <0.5 for yield of vines and total biomass, implying a significant role of non-additive genetic effect on these traits (Table 6).

**Table 6:** **Summary mean squares and significant tests of combining abilities and maternal effects for canopy temperature, canopy wilting, yield of storage root, skin color, flesh color, vine yield, total biomass and dry matter content of storage roots of sweetpotato clones evaluated across three sites in Rwanda**.

**Source of variation**	**DF**	**CT**	**CW**	**Storage root yield**	**Skin color**	**Flesh color**	**Vine yield**	**Total biomass**	**DMC**
GCA	7	4444.38^**^	131.99^**^	2318.59^**^	155.11^**^	30.41^**^	7444.48^**^	14929.05^**^	6221.73^**^
SCA	28	13937.89^**^	461.97^**^	14815.47^**^	941.52^**^	157.68^**^	129661.03^**^	218139.88^**^	36359.65^**^
REC	28	13276.30^**^	388.49^**^	3400.19^**^	359.96^*^	43.23^**^	37752.31^**^	53027.60^**^	33650.66^**^
MAT	7	2813.66^**^	95.65^**^	312.33 ns	50.59	4.67^**^	7168.89^*^	8215.46^*^	4113.67^**^
NMAT	21	10462.64^**^	292.84^**^	3087.86^*^	309.37^*^	38.56^**^	30583.43^**^	44812.14^**^	29537.00^**^
GCA × ENV	14	314.78	16.49	287.04^ns^	169.03^ns^	5.76^ns^	13650.82^**^	12967.79^ns^	2540.2^ns^
SCA × ENV	56	377.05	57.29	4630.03^ns^	530.24^ns^	29.69^**^	60392.33^**^	72456.75^**^	9933.92^**^
REC × ENV	56	736.71	55.83	3357.9^ns^	568.7^ns^	26.53^**^	40010.87^**^	49556.99^**^	9595.56^**^
MAT × ENV	14	163.89	22.58	737.79^ns^	148.5^ns^	6.64^*^	14262.23^**^	17174.70^**^	2834.87^ns^
NMAT × ENV	42	572.82	33.25	2620.11^ns^	420.2^ns^	19.89^**^	25748.64^*^	32382.29^ns^	6760.70^**^
GCA/SCA		0.72	0.7	0.56	0.57	0.61	0.31	0.35	0.58

*DF, degrees of freedom; CT, canopy temperature; CW, canopy wilting; DMC, dry matter content; GCA, general combining ability; SCA, specific combining ability; MAT, maternal effect; NMAT, non-maternal effect*;

** *Significant at p < 0.01*;

* *Significant at p < 0.05 (F-probability*;

ns *Not significant)*;

*REC, reciprocal crosses; ENV, environment; DMC, dry matter content*.

The reciprocal crosses showing maternal effects were significant (*P* ≤ 0.05) for CT, CW, flesh color and DMC of storage roots, vine yields and total biomass (Table 6). The reciprocal crosses had significant (*P* ≤ 0.05) differences for CT and WT, yields of storage roots and vines, flesh and skin color, total biomass and DMC of storage roots (Table 6). The GCA effects and sites had a highly significant interaction (*P* ≤ 0.01) for vine yield (Table 6). Likewise, the SCA effects of crosses and sites had significant interactions for flesh color, yield of vines, total biomass, and DMC of storage roots (Table 6). Maternal effects and sites showed significant interaction in influencing flesh color of storage roots, yields of vines and total biomass (Table 6).

#### General combining ability (GCA) effects

Negative GCA effects were estimated for the following parents: 8-1038 (−4.05), Otada 24 (−1.88) and 4-160 (−0.50) for canopy temperature and 8-1038 (−0.74), Otada 24 (−0.18), Ukerewe (−0.10) and 4-160 (−0.04) for canopy wilting, which are in a desirable direction for selection (Table 7). The highest GCA effects for yields of storage roots were 0.91, 0.81, and 0.48 for the parental genotypes, Nsasagatebo, K513261 and Ukerewe, respectively (Table 7). The genotypes that revealed the highest GCA effects for dry matter content were Nsasagatebo (3.12), 2005-034 (2.90) and Ukerewe (0.67) (Table 7). The highest GCA effects of 2.86, 2.36, and 1.97 for vine yields were observed in the parent's Nsasagatebo, 8-1038 and K513261, respectively. Likewise, the highest GCA effects of 3.71, 2.69, and 1.94 for total biomass were recorded for the genotypes Nsasagatebo, K513261 and Ukerewe, respectively (Table 7).

**Table 7:** **Estimates of GCA effects for canopy temperature, canopy wilting, yield of storage roots, skin color, flesh color, vine yield, total biomass and dry matter content of eight sweetpotato parents**.

**Genotype**	**CT**	**CW**	**Storage root yield**	**Skin color**	**Flesh color**	**Vine yield**	**Biomass**	**DMC**
2005-020	0.22^ns^	0.03^ns^	−0.95^*^	−0.21^ns^	0.01^ns^	−1.52^ns^	−2.50^ns^	−0.19^ns^
2005-034	0.89^**^	0.45^**^	−1.81^*^	0.10^ns^	−0.07^ns^	−4.25^*^	−6.13^**^	2.90^**^
4-160	−0.50^*^	−0.04^ns^	−1.03^ns^	−0.10^ns^	0.07^ns^	−1.30^ns^	−2.33^ns^	−1.16^ns^
8-1038	−4.05^**^	−0.74^**^	−0.85^ns^	−0.40^ns^	0.18^**^	2.36^ns^	1.53^ns^	−1.68^*^
K513261	1.83^**^	0.25^**^	0.81^ns^	−0.04^ns^	−0.11^*^	1.97^ns^	2.69^ns^	0.40^ns^
Nsasagatebo	2.38^**^	0.18^*^	0.91^ns^	0.41^ns^	0.02^ns^	2.86^ns^	3.71^ns^	3.12^**^
Otada 24	−1.88^**^	−0.18^*^	−1.28^*^	−0.67^**^	−0.27^*^	−3.42^ns^	−4.74^*^	−4.50^**^
Ukerewe	0.62^**^	−0.10^ns^	0.48^ns^	0.43^ns^	−0.09^ns^	1.60^ns^	1.94^*^	0.67^*^

*CT, canopy temperature; CW, canopy wilting; DMC, dry matter content*;

** *Significant at p < 0.01*;

* *Significant at p < 0.05*;

ns *Not significant)*.

#### Specific combining ability (SCA) and maternal effects

The SCA of direct crosses and reciprocals are presented in Table 8. The highest negative SCA effects of −13.26, −12.48, −11.61, −10.35 for canopy temperature were observed in the following crosses: Nsasagatebo × Otada 24, 2005-034 × 8-1038, 8-1038 × Nsasagatebo, Otada 24 × 4-160, respectively (Table 8). For canopy wilting, significantly negative SCA effects were recorded in Nsasagatebo × Otada 24 (−2.31), 8-1038 × Nsasagatebo (−2.06), 2005-034 × 8-1038 (−1.96), 4-160 × 2005-020 (−1.88) and Otada 24 × 4-160 (−1.54) (Table 8). Positive and high SCA effects of 15.75, 9.51, 8.92, 7.86, 6.15, 5.69, and 5.37 were observed for storage root yield in the crosses of 8-1038 × K513261, 4-160 × 8-1038, 2005-034 × 4-160, K513261 × Nsasagatebo, Otada 24 × Ukerewe, Nsasagatebo × Otada 24, 2005-020 × 2005-034, respectively (Table 8). The following crosses: Nsasagatebo × Otada 24, 8-1038 × K513261, 2005-034 × 4-160, 4-160 × 8-1038, expressed the highest positive SCA effects of 55.02, 48.44, 27.69, and 23.64, respectively, for vine yields. Similarly, the highest SCA effects for dry matter content were generated in the crosses of Nsasagatebo × Otada 24 (19.29), Otada 24 × 8-1038 (16.85), 4-160 × 8-1038 (14.92), Nsasagatebo × 2005-020 (14.87), 2005-034 × 4-160 (12.68), Otada 24 × 4-160 (9.37) and 4-160 × K513261 (9.05) (Table 8).

**Table 8:** **Estimates of SCA and maternal effects for canopy temperature, canopy wilting, yield of storage roots, vine yield and dry matter content of sweetpotato genotypes derived from direct and reciprocal crosses of eight parents**.

**Crosses**	**Traits**									
	**CT**		**CW**		**Storage root yield**		**Vine yield**		**DMC**	
	**Direct**	**Reciprocal**	**Direct**	**Reciprocal**	**Direct**	**Reciprocal**	**Direct**	**Reciprocal**	**Direct**	**Reciprocal**
2005-020 × 2005-034	−1.93^*^	0.28^ns^	−0.69^*^	−0.49^*^	5.37^*^	1.94^ns^	7.46^ns^	8.33^ns^	−1.36^ns^	1.99^ns^
2005-020 × 4-160	2.32^**^	−9.48^**^	−0.38^ns^	−1.88^**^	1.30^ns^	2.09^ns^	1.57^ns^	−5.84^ns^	1.45^ns^	−2.34^ns^
2005-020 × 8-1038	−4.39^**^	−1.06^ns^	−0.57^**^	−0.13^ns^	1.44^ns^	1.08^ns^	11.51^*^	5.16^ns^	4.83^**^	−1.36^ns^
2005-020 × K513261	−1.14^ns^	0.52^ns^	−0.20^ns^	−0.75^**^	−3.07^ns^	−1.48^ns^	−11.29^*^	−8.37^ns^	−0.82^ns^	−1.38^ns^
2005-020 × Nsasagatebo	0.18^ns^	−1.19^ns^	0.21^ns^	−0.36^ns^	−1.94^ns^	1.69^ns^	−9.95^ns^	7.64^ns^	−0.78^ns^	14.87^**^
2005-020 × Otada 24	3.71^**^	−0.81^ns^	0.69^**^	0.25^ns^	−1.18^ns^	−0.02^ns^	−6.27^ns^	−3.83^ns^	−9.02^**^	0.98^ns^
2005-020 × Ukerewe	−0.56^ns^	−0.08^ns^	0.70^**^	−0.27^ns^	−0.95^ns^	−2.47^ns^	9.05^ns^	0.81^ns^	0.89^ns^	−0.51^ns^
2005-034 × 4-160	3.32^**^	0.00	0.94^**^	0.00	8.92^**^	0.00	27.69^**^	0.00	12.68^**^	0.00
2005-034 × 8-1038	−2.48^**^	0.35^ns^	−1.96^**^	0.08^ns^	−4.08^*^	1.21^ns^	−22.64^**^	2.11^ns^	−22.65^**^	1.30^ns^
2005-034 × K513261	2.35^**^	0.31^ns^	0.32^ns^	0.23^ns^	0.98^ns^	1.28^ns^	8.39^ns^	14.63^*^	6.87^**^	−0.07^ns^
2005-034 × Nsasagatebo	2.21^**^	−0.23^ns^	0.13^ns^	0.03^ns^	2.03^ns^	−0.53^ns^	17.91^**^	−2.04^ns^	6.49^**^	4.91^*^
2005-034 × Otada 24	6.32^**^	10.99^**^	1.62^**^	1.56^**^	−1.15^ns^	−1.91^ns^	−11.55^*^	−10.08^ns^	1.21^ns^	−16.49^**^
2005-034 × Ukerewe	−6.16^**^	−1.00^ns^	−1.04^**^	−0.15*ns*	−3.50^*^	2.51^ns^	−11.81^*^	4.84^ns^	−8.5^**^	4.45^*^
4-160 × 8-1038	12.29^**^	−1.37^**^	1.85^**^	−1.04^**^	9.51^**^	0.38^ns^	23.64^**^	5.66^ns^	14.92^**^	0.38^ns^
4-160 × K513261	5.44^**^	0.47^ns^	0.45^*^	0.02^ns^	−0.15^ns^	−1.79^ns^	−5.60^ns^	−4.71^ns^	9.05^**^	−0.17^ns^
4-160 × Nsasagatebo	6.87^**^	9.86^**^	1.30^**^	1.53^**^	−0.45^ns^	−4.67^*^	−2.42^ns^	−18.08^**^	4.73^**^	−17.54^**^
4-160 × Otada 24	−1.24^*^	−10.35^**^	−0.29^ns^	−1.54^**^	0.84^ns^	2.67^ns^	−2.44^ns^	−5.07^ns^	−1.78^ns^	9.37^**^
4-160 × Ukerewe	−3.26^**^	10.46^**^	−0.36^ns^	1.77^**^	−0.74^ns^	−1.52^ns^	3.38^ns^	−18.68^**^	1.85^ns^	−13.10^**^
8-1038 × K513261	0.70^ns^	9.63^**^	0.95^**^	1.11^**^	15.75^**^	−3.27^ns^	48.44^**^	−10.38^ns^	3.94^ns^	−15.99^**^
8-1038 × Nsasagatebo	−11.61^**^	0.18^ns^	−2.06^**^	−0.26^ns^	−4.41^**^	2.33^ns^	−16.03^**^	−5.25^ns^	−13.02^**^	1.48^ns^
8-1038 × Otada 24	3.04^**^	−1.92^**^	0.78^**^	−0.44^ns^	−2.23^ns^	2.26^ns^	−10.69^*^	11.20^ns^	−2.58^ns^	16.85^**^
8-1038 × Ukerewe	1.21^*^	0.01^ns^	−0.30^ns^	0.43^ns^	−4.99^**^	0.74^ns^	−13.96^**^	1.00^ns^	−9.70^**^	1.11^ns^
K513261 × Nsasagatebo	−0.64^ns^	−0.11^ns^	0.25^ns^	−0.07^ns^	7.86^**^	−2.78^ns^	2.10^ns^	−17.45^**^	2.30^ns^	0.34^ns^
K513261 × Otada 24	3.13^**^	−0.69^ns^	0.78^**^	−0.48^*^	−0.44^ns^	−0.30^ns^	2.36^ns^	−10.21^ns^	3.64^**^	0.03^ns^
K513261 × Ukerewe	−0.54^ns^	0.69^ns^	−0.46^*^	0.28^ns^	0.05^ns^	1.69^ns^	10.08^ns^	5.99^ns^	2.19^ns^	8.44^**^
Nsasagatebo × Otada 24	−13.26^**^	−0.03^ns^	−2.31^**^	−1.22^**^	5.69^*^	−9.22^**^	55.02^**^	−2.92^ns^	19.29^**^	−5.70^**^
Nsasagatebo × Ukerewe	3.73^**^	10.43^**^	0.09^ns^	1.38^**^	4.99^**^	−0.93^ns^	−9.38^ns^	−1.28^ns^	0.46^ns^	−10.02^**^
Otada 24 × Ukerewe	2.91^*^	0.23^ns^	0.66^*^	−0.29^ns^	6.15^**^	−1.50^ns^	15.11^*^	1.90^ns^	8.31^**^	−1.51^ns^

*CT, canopy temperature; CW, canopy wilting; DMC, dry matter content*;

** *Significant at p < 0.01*;

* *Significant at p < 0.05*;

ns *Not significant*.

The reciprocal crosses showing maternal effects were significant, affecting the success rate of crosses. The direct crosses involving Otada 24 × 2005-034 and Ukerewe × Nsasagatebo were incompatible, while the corresponding reciprocal crosses were compatible (Table 3). The maternal effects were significant on CT, CW, storage root yields, flesh color, vine yields, total biomass and dry matter content of storage roots (Table 6). Several direct and reciprocal crosses revealed varied SCA effects (Table 8).

## Discussion

### Success rate of crosses, seed set and germination

The self- and cross-incompatibility of sweetpotato remains a major impediment for sweetpotato breeding (Martin, 1965, 1970; Kobayashi et al., 1993). Gasura et al. (2010) observed that some sweetpotato clones can be crossed easily. However, some female parents are difficult to cross with specific male parents. The same results were observed in this study. Complete incompatibility of both direct and reciprocal crosses was observed in seven pairs while 11 crosses showed partial incompatibility (Table 3). This result suggests that the success of genetic improvement of sweetpotato depends on an efficient selection of compatible parents.

The success rate of crosses varied from 3.7 to 66.7% with a mean of 19.3% (Table 3). According to Lebot (2009) the success rates of crosses depend on various factors such as compatibility, vigor of parents and weather conditions. According to Jones and Deonier (1965) and Jones et al. (1986) each capsule of sweetpotato has a maximum of 4 seeds, often with 1 to 2 seeds. Hand pollinated flowers produced up to 2 and rarely 3 seeds per capsule while insect pollinated flowers produced 3 to 4 seeds per capsule (Gasura et al., 2010). Lebot (2009) reported that about 50% of hand pollinated flowers produced two seeds. Similar results were observed in this study in which the number of seeds per capsule varied from 1 to 3 with a mean of 2 (Table 3).

Sweetpotato seed germination is irregular because of the hard seed coat (Miller, 1937). Chemical and mechanical scarification has been recommended to overcome this challenge (Wilson et al., 1989). In this study, variable seed germination was observed after scarification ranging from 10 and 85.1% with a mean of 43.8% (Table 3). Preliminary tests have shown that seeds that float in water germinate poorly. Most of the seed that sank was reportedly viable (Martin, 1946).

### Performance of newly developed families and parents

Previous reports pointed out highly significant (*P* < 0.001) effects of environment, genotype and genotype by environmental interactions on qualitative and quantitative traits of sweetpotatoes (Mwololo et al., 2009; Adebola et al., 2013; Kathabwalika et al., 2013). In the present study significant interactions between family and site were observed affecting yields of storage roots and vines, total biomass and DMC of storage roots of sweetpotato. The family effects were significant for all parameters evaluated, while site effects were significant for yields of storage roots and vines, total biomass and DMC of storage roots (Table 4). Kathabwalika et al. (2013) reported that the effects of genotypes, environments and their interactions contributed to 43.4, 34.8, and 21.8%, of the variation in storage root yields, respectively. Likewise, the strong contribution of genotype has been observed in the performance of sweetpotato genotypes (Nedunchezhiyan et al., 2007; Chataika et al., 2010). The highest proportion of the sum of squares of families for all evaluated traits indicated the existence of considerable genetic variation among the newly developed clones (Table 4).

### Drought tolerance

During the study period the Karama, Masoro and Rubona sites received 567.9, 722.4, and 804.3 mm rainfall, respectively (Table 2). The Karama site had lower rainfall than the minimum required for sweetpotato production which is about 600 mm. No supplemental irrigation was applied at this site. The trial was conducted under field conditions which had variable soil water content. These conditions could allow evaluation of drought tolerance of the test genotypes. A positive and significant correlation between yield and canopy temperatures were observed under drought stress conditions (Royo et al., 2002; Guendouz et al., 2012). This suggested that canopy temperature could be regarded as a valuable parameter to identify drought tolerance of crop genotypes. The canopy temperatures of wheat genotypes grown under well-watered conditions and drought stressed conditions was significantly different (Guendouz et al., 2012). In this study, families showed variation in canopy temperatures ranging from 16.9 to 22.7°C. The canopy temperatures found in this study were lower than those reported by Guendouz et al. (2012) under irrigated conditions (23.8 to 28.0°C) and under water stressed conditions (27.0 to 30.7°C). Canavar (2013) reported that canopy temperature is lower and dependent on the ambient temperatures of the environment. Therefore, ambient temperatures of experimental sites are different and can provide variable canopy temperatures.

Genotypes with water stress tolerance express low canopy temperatures under water stressed conditions (Blum, 2011; Pathan et al., 2014). This was also found in the present study in which some of the selected genotypes for drought tolerance (Nsasagatebo, 4-160 and 8-1038) had the lowest canopy temperatures (Table 5). The families that showed the lowest canopy temperature (<20°C) were, 4-160 × Ukerewe, 4-160 × Nsasagatebo, 8-1038 × 4-160, 4-160 × 8-1038, 8-1038 × 2005-020, Nsasagatebo × Ukerewe, with canopy temperatures of 18.9, 19.2, 19.3, 19.4, 19.7, 19.8°C, respectively (Table 5). Canopy temperatures measured using infrared thermometer provided useful data in determining drought tolerance of sweetpotato clones under water limited conditions. Therefore, this parameter can be considered as a rapid approach to assess drought tolerance in crop plants. However, other complementary techniques and parameters such as leaf water potential, canopy wilting, stomatal conductance, canopy senescence which are not plant destructive approaches and yield potential should be measured for efficient screening of crop genotypes for drought tolerance (Canavar, 2013).

Canopy wilting is the first visible symptom of water stress (Carter et al., 2006; Pathan et al., 2014). A slow canopy wilting and minimal yield reduction under drought stress are important traits that should be evaluated to determine drought tolerance in crop genotypes (Pathan et al., 2014). For example, in soybean, slow canopy wilting and sustained nitrogen fixation under drought stress have resulted in yield increases in water-limited environments (Sinclair et al., 2007). In this study, the lowest mean canopy wilting scores of 0.86, 1.09, 1.19, 1.33, 1.33, 1.47, 1.50 were observed among the crosses of 4-160 × Nsasagatebo, 4-160 × Ukerewe, Otada 24 × 4-160, Nsasagatebo × 2005-020, Otada 24 × Nsasagatebo, 4-160 × K513261 and K513261 × 4-160, respectively (Table 5). Among parents, Nsasagatebo developed the lowest level of canopy wilting (1.1), followed by 4-160 (1.5) and 8-1038 (1.6). Based on low canopy temperature and canopy wilting, the following families were ranked and selected: 4-160 × Nsasagatebo, 4-160 × Ukerewe, Otada 24 × 4-160, Nsasagatebo × 2005-020, Otada 24 × Nsasagatebo, 4-160 × K513261, 513261 × 4-160, 8-1038 × 4-160, 4-160 × 8-1038, 8-1038 × 2005-020 and Nsasagatebo × Ukerewe.

### Yield of storage roots

Sites had significant effects on the yield of storage roots of sweetpotato clones. Previous reports showed that the average storage root yield was higher in the Nairobi region (16.8 t ha^−1^) than in Western Kenya (15.2 t ha^−1^) (Mwamburi and Ndolo, 2013). Traits associated with storage root yields such as size of storage roots and number of storage roots per plant have been reported to be strongly affected by changes in environmental conditions (Nedunchezhiyan et al., 2007). In this study, sites caused variation in the storage root yields. The highest mean storage root yield of 6.9 t ha^−1^ was observed at Rubona, followed by Karama with 5.5 t ha^−1^ and Masoro with 4.5 t ha^−1^ (Table 5). In Uganda Gasura et al. (2010) reported three classes; high yielding (18-30 t ha^−1^), moderately yielding (11-17 t ha^−1^) and low yielding (<11 t ha^−1^) sweetpotato genotypes. Storage root yields ranging between 63.3 and 22.1 t ha^−1^ have been reported in South Africa (Adebola et al., 2013). The storage root yields found in this study were much lower than in these reports. The tested clones are in the early selection phase and their yield response could be affected by their genetic constitution and the environment. This requires continuous selection of genetically fixed, stable and high yielding clones across representative sites in Rwanda. Moreover, fertilizers, pesticides and irrigation were not applied in this study, whereas they were in the other trials such as reported by Adebola et al. (2013).

### Dry matter content of storage roo Ts

The dry matter content of sweetpotato storage roots is influenced by site and genotype effects (Shumbusha et al., 2010). Variable DMC has been reported in Kenya ranging from 23.5 to 34.5% (Kivuva et al., 2015). According to the 2014 catalog of Orange-Fleshed Sweetpotato for Africa, the orange fleshed sweetpoaoto genotypes KENSPOT-3 and KENSPOT-5 had DMC of 32.5% and 25.9%, in that order. Significant effects of site and genotype on DMC were observed in the present study. The highest DMC (28.3%) was observed at Rubona followed by Masoro (26.3%) and Karama (22.6%) (Table 5).

In another study, DMC of sweetpotato storage roots varied from 25.6 to 33.3% among families, while these values were 25.3 to 45.4% for individual clones (Courtney et al., 2008). In the present study, DMC among families varied from 25.9 to 37.5% (Table 5). The best families in DMC across site were, 8-1038 × K513261, Nsasagatebo × 8-1038, Otada 24 × 4-160, Nsasagatebo × Ukerewe and 4-160 × Nsasagatebo. These families had DMC of 37.5, 37.2, 35.7, 35.3, and 35.1% in the storage roots, respectively (Table 5). These families are promising for future release, by showing DMC of >30%, an important attribute for farmer's adoption of new sweetpotato varieties in Rwanda. Therefore, further evaluations across representative growing environments are needed to identify their adaptability and stability.

### Heritability

Genetic improvement of crop plants depends on the magnitude of heritability of economic traits (Maluf et al., 1983; Ma-Teresa et al., 1994). Previous results recorded heritability of 0.93 for DMC of sweetpotato storage roots among full-sibling families (Courtney et al., 2008). Heritability of 0.11 to 0.75 was reported for root yield, 0.07 to 0.75 for root size, and 0.26 to 0.50 for dry matter (Ma-Teresa et al., 1994). Heritability of non-marketable roots was 0.6 (Maluf et al., 1983). The present study found heritability of 0.95, 0.84, 0.68, 0.47, 0.74 and 0.75, 0.50 and 0.58 for canopy temperature, canopy wilting, storage root yield, skin color, flesh color, DMC, yield of vines and total biomass, respectively (Table 4). Some of these estimates agree with previous findings. According to Courtney et al. (2008), heritability differs from one population to another, and with the test environment. Heritability for nutrient composition may vary due to soil nutrients such as the macro- and micro-elements. High heritability estimates indicate a higher frequency of genes controlling the traits (Ma-Teresa et al., 1994) and the potential to improve these traits with traditional breeding strategies (Courtney et al., 2008; Mwije et al., 2014). Accordingly, heritability observed for canopy temperature, canopy wilting, yield of storage roots and DMC of storage roots indicate that the genetic improvement of these traits can be achieved through conventional breeding.

### General and specific combining ability effects

The GCA and SCA analysis revealed significant differences (*P* < 0.01) among genotypes for canopy temperature, canopy wilting, yield of storage roots and vines, total biomass and DMC of storage roots (Table 6). Saad (1993) reported that effects of GCA and SCA were significant for yield, storage root number and mean root weight. GCA and SCA mean squares for flesh yield and root DMC were highly significant (Chiona, 2009). Previous diallel analysis revealed significant GCA and SCA effects in the study of heritability of putative drought adaptation traits in sweetpotato (Mwije et al., 2014).

The GCA/SCA ratio was > 50% for canopy temperature, canopy wilting, storage root yields, skin color, flesh color and DMC of storage roots but not for vines yield and total biomass (Table 6). These results agree with the findings of Chiona (2009) who reported that the ratio of GCA/SCA for storage root yield was 0.68. Baker (1978) indicated that high ratios of GCA/SCA mean that the additive gene action makes a greater contribution to the expression of specific traits than non-additive gene action. This study revealed that the additive gene action had important effects in expression of canopy temperature, canopy wilting, storage root yield, skin color, flesh color and DMC of storage roots, while the non-additive gene action had significant effects in the expression of vine yields and total biomass.

In the drought tolerance studies, genotypes that presented the highest negative general combining abilities for canopy temperature and canopy wilting were the most desirable. These genotypes were 8-1038 (−4.05), Otada 24 (−1.88) and 4-160 (−0.50) for canopy temperature and 8-1038 (−0.74), Otada 24 (−0.18), Ukerewe (−0.10) and 4-160 (−0.04) for canopy wilting (Table 7). The selection of parents based on their combining ability, and understanding the genetic control of key traits ensure the efficiency of a breeding programme (Nadarajan and Gunasekaran, 2005; Sleper and Poehlman, 2006). In the current study, good general combiners for drought tolerance were the parents, 8-1038, Otada 24 and 4-160. These genotypes revealed the lowest canopy temperature and wilting. Good combiners for high storage root yields were Nsasagatebo, K513261 and Ukerewe, while good combiners for high DMC were, Nsasagatebo, 2005-034 and Ukerewe (Table 7).

Specific combining ability effects are useful to identify specific crosses with desirable traits (Acquaah, 2007). In this study, the best specific crosses for drought tolerance were Nsasagatebo × Otada 24, 2005-034 × 8-1038, 8-1038 × Nsasagatebo and Otada 24 × 4-160. These had the lowest canopy temperature and wilting level (Table 8). The crosses of 8-1038 × K513261, 4-160 × 8-1038, 2005-034 × 4-160, K513261 × Nsasagatebo, Otada 24 × Ukerewe, Nsasagatebo × Otada 24 and 2005-020 × 2005-034 were selected for their high storage root yields. The best crosses for high DMC were Nsasagatebo × Otada 24, Otada 24 × 8-1038, 4-160 × 8-1038, Nsasagatebo × 2005-020, 2005-034 × 4-160, Otada 24 × 4-160 and 4-160 × K51326 (Table 8).

### Maternal effect

Maternal effects are common in sexually reproducing crops, and these can be detected by measuring the genetic differences of individuals arising from direct and reciprocal crosses (Grami and Stefansson, 1977). A trait is controlled by nuclear genes when the direction of cross did not affect its quantity and quality of expression (Gedye et al., 2005). Lin et al. (2007) reported maternal effects on yields of storage roots and vines in Clone I selections of sweetpotato. In the current study the maternal effects affected the compatibility between genotypes where partial compatibility was observed in the crosses of Otada 24 × 2005-034 and Ukerewe × Nsasagatebo (Table 3). The maternal effects were significant among families for canopy temperature, canopy wilting, flesh color, DMC, yield of vines and total biomass. This was confirmed by the significant effects of reciprocal crosses and their varied SCA effects (Tables 6, 8). The existence of maternal effects is important for sweetpotato breeders in considering the direction of crosses to be performed to improve a particular trait.

The present study examined combining abilities, maternal effects and heritability of drought tolerance, yield and yield components in newly developed sweetpotato clones. In some direct and reciprocal crosses complete incompatibility was observed suggesting that the success of genetic improvement of sweetpotato depends on an efficient selection of compatible parents. High levels of broad sense heritability and significant GCA and SCA effects detected for canopy temperature, canopy wilting, storage root yields and DMC of storage roots indicated that these traits can be improved through conventional breeding. The ratio of GCA/SCA >50% on canopy temperature, canopy wilting, yield of storage roots and DMC of storage roots revealed the predominance of additive gene action. The best general combiners were parents 8-1038, Otada 24 and 4-160 for drought tolerance; Nsasagatebo, K513261 and Ukerewe for high storage root yield; and Nsasagatebo, 2005-034 and Ukerewe for high DMC. Based on low canopy temperatures, low levels of canopy wilting and high storage root yields and DMC, the families selected for breeding or direct production were 2005-020 × 4-160, 2005-034 × 2005-020, 8-1038 × 4-160, 8-1038 × Ukerewe, Nsasagatebo × Otada 24, Otada 24 × 8-1038, Otada 24 × K513261, Ukerewe × 2005-034, and Ukerewe × 8-1038. The selected families are recommended for further evaluation to determine their yield potential and stability for release in Rwanda or similar environments.

## Author contributions

All authors listed, have made substantial, direct and intellectual contribution to the work, and approved it for publication.

### Conflict of interest statement

The authors declare that the research was conducted in the absence of any commercial or financial relationships that could be construed as a potential conflict of interest.
